# Comparison of daratumumab-based regimens as second-line therapy in relapsed/refractory multiple myeloma

**DOI:** 10.1038/s41408-023-00957-w

**Published:** 2023-12-11

**Authors:** Charalampos Charalampous, Utkarsh Goel, Prashant Kapoor, Moritz Binder, Francis K. Buadi, Joselle Cook, David Dingli, Angela Dispenzieri, Amie L. Fonder, Morie A. Gertz, Wilson Gonsalves, Suzanne R. Hayman, Miriam A. Hobbs, Yi L. Hwa, Taxiarchis Kourelis, Martha Q. Lacy, Nelson Leung, Yi Lin, Rahma Warsame, Robert A. Kyle, S. Vincent Rajkumar, Shaji K. Kumar

**Affiliations:** 1 Division of Hematology, Department of Internal Medicine, Mayo Clinic, Rochester, MN USA; 2 Division of Nephrology and Hypertension, Department of Internal Medicine, Mayo Clinic, Rochester, MN USA

**Keywords:** Myeloma, Risk factors

Dear Editor,

Multiple myeloma (MM) is a hematologic malignancy characterized by the proliferation of abnormal plasma cells in the bone marrow [1]. Despite the availability of various treatment options, MM remains an incurable disease, with frequent relapses necessitating ongoing therapy [2]. In recent years, the introduction of daratumumab (dara) has revolutionized the management of MM [3, 4]. Several clinical trials have evaluated daratumumab-based regimens in patients with relapsed/refractory MM, demonstrating significant improvements in survival rates [5, 6]. However, the optimal sequencing and combination of daratumumab-based regimens in relapsed/refractory MM remain unclear. Specifically, combinations of daratumumab with an immunomodulator (IMiD) or a proteasome inhibitor (PI) have been studied in this setting, but with no comparative studies to guide the clinician in terms of the ideal combination. In this retrospective study, we aim to compare the efficacy of different daratumumab-based regimens as second-line therapy in relapsed/refractory MM and provide a benchmark for the expected clinical outcomes of these patients.

We evaluated patients with relapsed/refractory MM from first-line therapy between 2016–2022, seen at Mayo Clinic, Rochester. All patients had to receive a daratumumab-based treatment as second-line therapy for relapsed/refractory MM, and patients who started daratumumab for other reasons (e.g., toxicity) were excluded. We grouped patients based on the dara combination as follows: dara-IMiD (lenalidomide, pomalidomide), dara-PI (bortezomib, ixazomib, and carfilzomib), dara monotherapy, and other combinations. Approval for this study was obtained from the Mayo Clinic Institutional Review Board. The mSMART classification was used for FISH risk stratification {t(4;14), t(14:16), t(14;20), deletion 17p, and gain/amplification 1q}.

The endpoints of the study were progression-free survival (PFS) and overall survival (OS); the Kaplan–Meier method was used for median estimates. Both PFS and OS were measured from the date of daratumumab initiation as second-line therapy. The Cox proportional hazard model was used to estimate hazard ratios (HR). A two-sided *p*-value of less than 0.05 was considered for statistical significance. All statistical analyses and graphs were performed using R.

A total of 404 patients met our criteria. The average age of diagnosis was 63.6 years (range: 27.1–90.4), and 63.1% were male. During induction treatment, the majority (94%) of patients were given a combination of three drugs (such as VRd or CyBord), and 5.7% were given a combination of two drugs (such as Rd or Vd). Nearly 70% of patients received an upfront autologous stem cell transplant.

At first relapse, 160 (39.6%) patients were refractory to an IMiD, with only two refractory to pomalidomide and the rest to lenalidomide. In addition, 81 (20%) patients were refractory to bortezomib, 12 (3%) were refractory to either carfilzomib or ixazomib, 75 (18.6%) were refractory to both an IMiD and a PI, and 76 (18.8%) were refractory to no drugs. For second-line treatment, 216 (53.5%) patients were given a combination of daratumumab with an IMiD, 140 (34.7%) were given a combination of daratumumab with a PI, 23 (5.7%) were given daratumumab alone, and 19 (4.7%) were given both an IMiD and a PI. More specifically, 110 (27.2%) had a lenalidomide-based regimen, 106 (26.2%) had pomalidomide, 106 (26.2%) had bortezomib, and 34 (8.4%) had either ixazomib or carfilzomib. Finally, 137 (33.9%) patients were given a drug to which they were already refractory in their first-line treatment.

The median PFS was 19.3 months (17.1–23.9 months), and the median OS was 71.1 months (59.9–NR months).

First, we compared patients treated with dara-IMiD and patients treated with dara-PI. The dara-IMiD combination resulted in an increase in median PFS (28.7 vs. 13.5 months, *p* < 0.01, respectively) compared to dara-PI (Fig. 1). When stratifying patients based on FISH, we found that both high-risk patients (25.3 vs. 8.1 months, *p* = 0.01, respectively) and standard-risk patients (45.5 vs. 17 months, *p* < 0.01, respectively) benefited significantly from the dara-IMiD regimen. For OS, no difference was seen between the dara-IMiD and dara-PI in our cohort (72.9 vs. 52.6 months, *p* = NS, respectively).

**Fig. 1:**
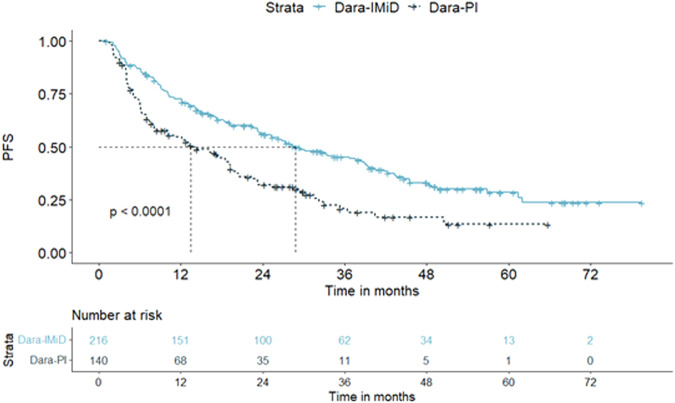
Progression-free survival (PFS) in patients treated with dara-IMiD vs. dara-PI for the whole cohort. PFS for dara-IMiD treated patients vs. dara-PI: 28.7 vs. 13.5 months, respectively. The advantage was consistent across all FISH risk categories.

We then compared patients treated with the dara-IMiD combination, and we found that patients who were already refractory to IMiDs from first-line had significantly shorter PFS compared to those who were not (14.6 vs. 43.3 months, *p* < 0.01, respectively). In addition, the lenalidomide-based combination resulted in significantly better PFS compared to pomalidomide (39.4 vs. 22.4 months, *p* < 0.01, respectively). However, the difference was not sustained when we looked at patients already refractory only to IMiDs from first-line (14.2 vs. 17.8 months, *p* = NS, respectively). Finally, we compared the dara-IMiD and the dara-PI combination in the IMiD refractory population, and we found no statistically significant differences between the two groups (16.2 vs. 12.8 months, *p* = NS, respectively) (Fig. 2).

**Fig. 2:**
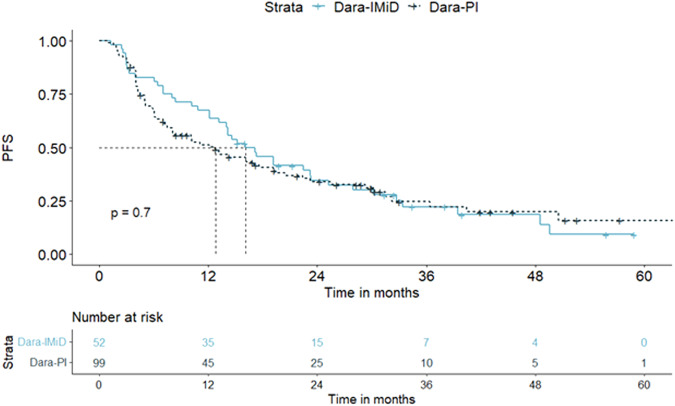
Progression-free survival (PFS) in patients treated with dara-IMiD vs. dara-PI for patients already refractory to IMiDs. No significant differences were seen between dara-IMiD vs. dara-PI in IMiD-only refractory patients, PFS: 16.2 vs.12.8 months, p = NS, respectively.

In the multivariable analysis for PFS using age, ISS, mSMART risk, and refractoriness to any drug when starting the second line regimen, the dara-PI combination resulted in an increased risk for earlier progression with a HR = 1.84 (1.36–2.49, *p* < 0.01) compared to the dara-IMiD combination (Table 1). Refractoriness to any drug increased the risk for progression in univariable analysis, HR = 1.75 (1.21–2.53, *p* < 0.01). However, the effect was mitigated with no statistically significant difference in the multivariable model, HR = 1.26 (95% CI: 0.82–1.92, *p* = NS).

**Table 1:** Multivariable Cox model in our cohort.

Variable	Value	*N*	Events	Hazard ratio	95% CI	*p*-value
Age	Older than 65 years	114	72	1	Reference	
	Younger than 65 years	179	113	1.12	0.82–1.51	0.47
ISS	1 OR 2	199	124	1	Reference	
	3	94	61	1.17	0.85–1.6	0.34
mSMART risk	Standard-risk	145	85	1	Reference	
	High-risk	148	100	1.44	1.06–1.95	**0.02**
Second-line therapy	IMiD	185	107	1	Reference	
	PI	108	78	1.84	1.36–2.49	**0.01**
Refractory to any drug	No	50	27	1	Reference	
	Yes	243	158	1.26	0.82–1.92	0.29

The dara-IMiD combination is independently associated with increased PFS in our cohort, even when adjusting for other risk factors, including refractoriness to any drug at first-line.

Bold values indicates statistical significant *P* values (*P* < 0.05).

Our findings show that patients treated with a combination of daratumumab and an IMiD had a better PFS compared to those treated with a combination of daratumumab and a PI. The benefit of the dara-IMiD regimen was also shown in a multivariable analysis. The synergistic effect of IMiDs and daratumumab is potentially due to the IMiD-mediated upregulation of CD38 expression and the subsequent priming of MM cells for anti-CD38 targeting [7]. The lenalidomide-based combination resulted in a better PFS compared to pomalidomide, except for patients already refractory to IMiDs from first-line treatment. There was no significant difference in PFS between the dara-IMiD and dara-PI combination in the IMiD refractory population.

While previous clinical trials have examined daratumumab in relapsed/refractory MM patients we only looked at daratumumab as second-line therapy [5, 6, 8–10]. We thus reduced the potential effect of prior treatment on dara-related outcomes. Moreover, this is a study with no exclusion criteria; therefore, these data can be used as a potential reference for expected outcomes. A recent study evaluated 583 MM patients treated with a dara-containing regimen, irrespective of the previous number of lines [11]. For the second line, they reported a median PFS of 23.5 months and OS of 49.1 months, similar to our experience. They also found that the dara-lenalidomide combination had the longest PFS compared to dara-pomalidomide and dara-bortezomib (26.8 vs. 9.7 vs. 8.3 months, respectively). Another study reported a median time to progression (TTP) of 10.8 months in the DVd group, while for the DRd group, the median TTP was not reached [12].

The POLLUX (DRd) and the CASTOR (DVd) trials reported a median PFS of 44.5 months and 16.7 months, respectively [13, 14]. Both trials excluded patients refractory to lenalidomide and bortezomib, respectively, which may explain the increased PFS in these trials compared to our study. Indeed, for patients not refractory to lenalidomide, we reported a median PFS of 43.3 months, similar to that of POLLUX. In a subgroup analysis of the CASTOR trial among patients with only one prior line of therapy, the median PFS was 27 months compared to the 13.5 months we reported in this study. The discrepancy could be potentially explained by the biases introduced by the selection criteria in randomized trials, which may preferentially select for “fitter” patients overall [15].

Our study has several limitations. The lack of randomization and knowledge of the factors affecting treatment decisions preclude any concrete conclusions for direct comparisons of the different regimens. Therefore, these findings can only be used for hypothesis generation but could only be confirmed in a randomized clinical trial setting.

In summary, our findings potentially suggest that the combination of daratumumab and an IMiD leads to an increased PFS compared to the combination of daratumumab and bortezomib. Carfilzomib could not be compared due to the low numbers in this study. From the dara-IMiDs, the DRd regimen seems more efficacious than DPd in patients not refractory to lenalidomide at the second line. Importantly, in patients refractory to IMiDs, no combination resulted in a significantly different PFS among the three most used regimens (DPd, DRd, DVd). Further studies are needed on this topic to address the best second-line combination for this population.

### Supplementary information

Supplemental material

## Data Availability

For original data, please contact kumar.shaji@mayo.edu.
