# Supplementary material for: Comparison of daratumumab-based regimens as second-line therapy in relapsed/refractory multiple myeloma
Source: Blood Cancer J. 2023 Dec 11;13(1):183. doi: 10.1038/s41408-023-00957-w (PMC10711005; doi:10.1038/s41408-023-00957-w)
Supplement: Supplementary file 1 — Supplemental material [file 41408_2023_957_MOESM1_ESM.docx]

**Supplementary material:**

**STable 1. Baseline and treatment characteristics of the entire cohort**

**SFigure 1. Progression-free survival (PFS) in patients treated with dara-IMiD vs. dara-PI for high-risk patients**

**SFigure 2. Progression-free survival (PFS) in patients treated with dara-IMiD vs. dara-PI for standard-risk patients**

**SFigure 3. Progression-free survival (PFS) comparison between patients treated with dara-IMiD at first relapse: IMiD-refractory versus not-IMiD refractory**

**SFigure 4. Progression-free survival (PFS) in patients refractory to lenalidomide vs. not refractory to lenalidomide at first relapse**

**Table 1. Baseline and treatment characteristics of the entire cohort**

All patients (N=404) were treated with a daratumumab-containing regimen as second-line therapy for relapsed and/or refractory multiple myeloma

|  | **Overall (N=404)** |
| --- | --- |
| **Age (years)** |  |
| Median (range) | 63.6 (17.1 – 90.4) |
| **Race** |  |
| White | 375 (93.5%) |
| Black | 8 (2.0%) |
| Asian | 7 (1.7%) |
| Other | 11 (2.7%) |
| **Sex** |  |
| Female | 149 (36.9%) |
| Male | 255 (63.1%) |
| **ISS** |  |
| 1 | 110 (30.8%) |
| 2 | 135 (37.8%) |
| 3 | 112 (31.4%) |
| **R-ISS** |  |
| 1 | 72 (21.4%) |
| 2 | 207 (61.4%) |
| 3 | 58 (17.2%) |
| **FISH** |  |
| Standard-Risk | 176 (47.2%) |
| High-Risk | 141 (37.8%) |
| Double-Hit | 56 (15.0%) |
| **ASCT as first-line** |  |
| No | 126 (31.2%) |
| Yes | 278 (68.8%) |
| **Number of drugs at first line** |  |
| Doublet | 23 (5.7%) |
| Triplet | 379 (93.8%) |
| Quadruplet | 1 (0.2%) |
| **Drug classes at second line** |  |
| Daratumumab | 23 (5.7%) |
| Dara-IMiD | 216 (53.5%) |
| Dara-PI | 140 (34.7%) |
| Both (IMiD and PI) | 19 (4.7%) |
| Other | 6 (1.5%) |
| **Drug classes at second line** |  |
| Lenalidomide | 110 (27.2%) |
| Pomalidomide | 106 (26.2%) |
| Bortezomib | 106 (26.2%) |
| Carfilzomib | 22 (5.4%) |
| Ixazomib | 12 (3.0%) |
| Both (IMiD and PI) | 19 (4.7%) |
| Other | 29 (7.2%) |
| **Drugs refractory to from first-line** |  |
| Lenalidomide | 160 (39.6%) |
| Bortezomib | 81 (20.0%) |
| Carfilzomib | 6 (1.5%) |
| Ixazomib | 6 (1.5%) |
| None | 76 (18.8%) |
| Both (IMiD and PI) | 75 (18.6%) |
| **Treatment with a drug already**  **refractory from first-line** |  |
| No | 267 (66.1%) |
| Yes | 137 (33.9%) |

Abbreviations: ASCT; autologous stem cell transplant, IMiD; immunomodulator, PI; proteasome inhibitor


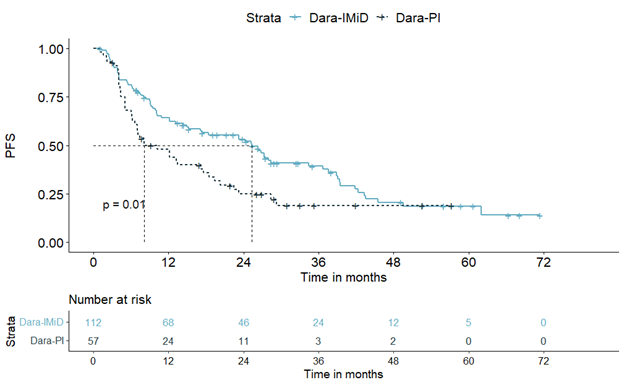


**SFigure 1. Progression-free survival (PFS) in patients treated with dara-IMiD vs. dara-PI for high-risk patients**


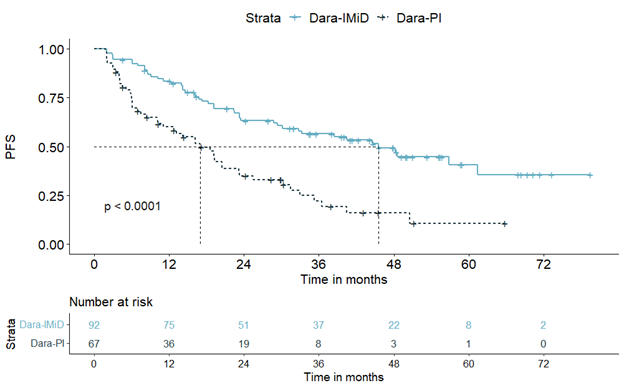


**SFigure 2. Progression-free survival (PFS) in patients treated with dara-IMiD vs. dara-PI for standard-risk patients**


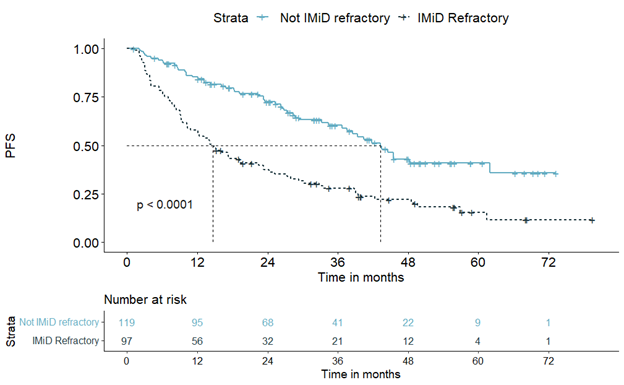


**SFigure 3. Progression-free survival (PFS) comparison between patients treated with dara-IMiD at first relapse: IMiD-refractory versus not-IMiD refractory**


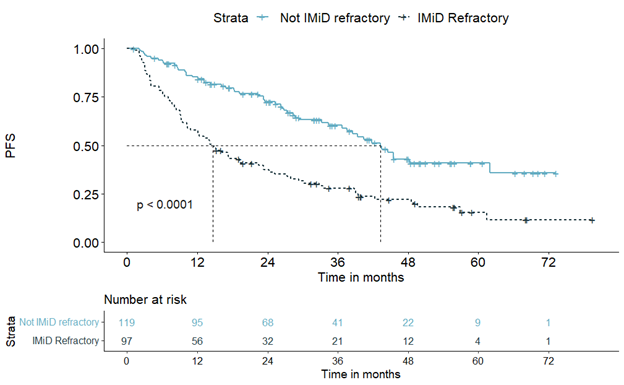


**SFigure 4. Progression-free survival (PFS) in patients refractory to lenalidomide vs. not refractory to lenalidomide at first relapse**
